# Normal range of complement components during pregnancy: A prospective study

**DOI:** 10.1111/aji.13202

**Published:** 2019-11-12

**Authors:** Ying‐dong He, Bing‐ning Xu, Di Song, Ya‐qin Wang, Feng Yu, Qian Chen, Ming‐hui Zhao

**Affiliations:** ^1^ Department of Obstetrics and Gynecology Peking University First Hospital Beijing China; ^2^ Key Laboratory of Renal Disease Ministry of Health of China Key Laboratory of Chronic Kidney Disease Prevention and Treatment Ministry of Education of China Beijing China; ^3^ Department of Medicine Renal Division Peking University First Hospital Peking University Institute of Nephrology Beijing China; ^4^ Peking‐Tsinghua Center for Life Sciences Beijing China

**Keywords:** complement system, normal pregnancy, normal range

## Abstract

**Problem:**

The complement system plays a key role in normal placentation, and delicate regulation of complement system activation is critical for successful pregnancy. Therefore, establishing a normal range of complement components during pregnancy is important for clinical evaluation and research.

**Methods:**

We performed a prospective study to investigate the normal range of complement components in circulation during different stages of pregnancy. Plasma concentrations of complement factor B (CFB), C1q, complement factor H (CFH), C3, C3c, and C4 were measured using an immunoturbidimetric assay; mannan‐binding lectin (MBL), C3a, C5a, and soluble C5b‐9 (sC5b‐9) levels at different time points of pregnancy were determined by enzyme‐linked immunosorbent assay (ELISA).

**Results:**

A total of 733 plasma samples were collected from 362 women with a normal pregnancy and 65 samples from non‐pregnant women. In the first trimester of pregnancy, the levels of CFB, CFH, MBL, C3c, C4, and C3a were 414.5 ± 85.9 mg/L (95% CI for mean: 402.4‐426.6 mg/L), 381.0 ± 89.0 mg/L (95% CI for mean: 368.5‐393.6 mg/L), 4274.5 ± 2752 ng/mL (95% CI for mean: 3881.1‐4656.4 ng/mL), 1346.9 ± 419.8 mg/L (95% CI for mean: 1287.7‐1406.0 mg/L), 357.4 ± 101.8 mg/L (95% CI for mean: 343.0‐371.7 mg/L), and 182.5 ± 150.0 ng/mL (95% CI for mean: 186.9‐229.1 ng/mL), respectively. The levels of C3 and C4 increased gradually throughout pregnancy. The levels of C1q, C5a, and sC5b‐9 in the first and second trimesters were nearly the same as those in non‐pregnant women.

**Conclusion:**

The results of this study show that pregnancy itself may influence the plasma levels of complement system components.

## 1 INTRODUCTION

In normal pregnancy, the maternal immune system undergoes certain changes, including downregulation of polymorphic classical class I human leukocyte antigen molecules (HLA‐A and HLA‐B) in invasive trophoblasts,^1^, ^2^ a shift from the T helper (Th) 1 to Th2 phenotype,^3^ and expression of uterine natural killer (uNK) cells at the maternal‐fetal interface^4^, ^5^ to protect the semiallogeneic fetus and placenta from attack. Recent studies have found that the complement system plays an important role in normal placentation and that delicate regulation of complement system activation is critical for successful pregnancy.^6^, ^7^

The complement system can be activated via three pathways: the classical pathway, alternative pathway, and mannan‐binding lectin (MBL) pathway. All three can lead to increased synthesis of C3 and C5 convertases, resulting in the cleavage of C3 and C5 to produce C3a and C5a, respectively. Activation of C5 may further stimulate the terminal pathway of the complement system to generate the membrane attack complex (MAC). C1q is the core molecule in the classical pathway of complement activation and is widely distributed in the human decidual stroma.^8^ Normal C1q expression at the maternal‐fetal interface plays an important role in placental formation and pregnancy maintenance.^8^, ^9^, ^10^ Complement factor B (CFB) can bind to C3b to form C3 convertase, promoting activation of the complement system via the alternative pathway, and this process can be inhibited by complement factor H (CFH), an inhibitor of the alternative pathway. The lectin pathway activates the complement system via MBL‐associated serine proteases (MASPs). In both the classical and lectin pathways, C3 convertase is produced via the cleavage of C4, and the plasma level of C4 reflects the activation potential of these two pathways. Many apoptotic cells and free deoxyribonucleic acid (DNA) are produced during placental formation in early pregnancy; such cellular debris and DNA fragments can lead to complement activation and thus complement system‐mediated clearance.^6^, ^7^ However, the process of physiological placentation can be assured by three complement regulatory proteins expressed in syncytiotrophoblasts: decay‐accelerating factor (DAF), membrane cofactor protein (MCP), and CD59.^11^, ^12^

In general, moderate activation of the complement system is crucial for maintaining pregnancy, and any deviation from normal activation and regulation of the complement system may result in adverse pregnancy outcomes, such as recurrent spontaneous abortion,^13^ preterm birth,^14^, ^15^, ^16^ and preeclampsia.^17^, ^18^, ^19^, ^20^, ^21^, ^22^, ^23^ Therefore, establishing a normal range of complement components during pregnancy is important for clinical evaluation and research. In addition, changes in circulating complement components may provide accessible biomarkers for predicting adverse pregnancy outcomes if “normal” pregnancy components are understood. Unfortunately, almost all previous clinical studies involving dysregulation of the complement system and adverse pregnancy outcomes were cross‐sectional in design. In this study, we attempted to determine the normal range of complement components in circulation during different stages of normal pregnancy.

## 2 MATERIALS AND METHODS

### 2.1 Patient enrollment

Pregnant women who visited Peking University First Hospital for prenatal care between April 1st, 2014, and December 31st, 2015, were screened for this prospective study. Color Doppler ultrasound was performed at 6‐8 weeks after the last menstrual period to assess gestational age. A medical history was also collected, and a physical examination and blood tests were performed to determine whether the women were eligible to participate in this study. Those with any disease prior to pregnancy were excluded. A total of 488 eligible patients who agreed to participate were enrolled in this study. The patients were followed up for their pregnancy outcomes.

**Figure 1:**
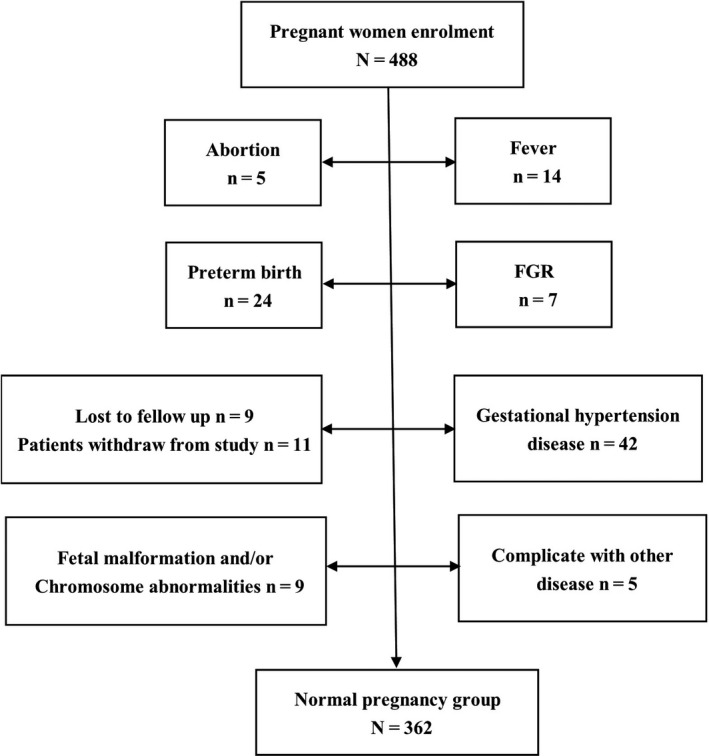
Patient enrolment

Among the 488 pregnant women enrolled, 42 suffered from gestational hypertension disease, 5 had spontaneous abortions, 24 had premature births, 9 had fetal malformations and/or chromosomal aberrations, 7 had fetal growth retardation (FGR), 14 suffered from fever during pregnancy, 3 had thrombocytopenia during the third trimester, 1 had intrahepatic cholestasis, 1 had a placental abruption, 9 were lost to follow‐up, and 11 withdrew from the study. Thus, a total of 362 gravidas were regarded as the normal pregnancy group and additional 65 healthy, non‐pregnant women were included in the non‐pregnancy group as normal controls. To investigate the plasma levels of complement components at different stages of pregnancy, we divided the pregnancy continuum into five stages at intervals of 7 weeks: 6‐12^+6^ weeks; 13‐19^+6^ weeks; 20‐27^+6^ weeks; 28‐35^+6^ weeks; and 36^+^ weeks. The procedure for patient enrollment is shown in Figure 1.

### 2.2 Diagnostic criteria

Premature birth^24^ and FGR^25^ were diagnosed by the obstetricians according to the American College of Obstetricians and Gynecologists (ACOG) guidelines. Gestational hypertension and preeclampsia were diagnosed according to the relevant ACOG (2013) guidelines.^26^ Fever was considered to exist when the temperature was ≥37.8°C for any reason during pregnancy.

### 2.3 Plasma preparation and laboratory assessment

Peripheral blood samples were collected from the participants every 6‐8 weeks during pregnancy. Blood samples were obtained from an antecubital vein and drawn directly into EDTA‐K_2_ anticoagulation tubes and then centrifuged within 1 hour at a relative centrifugal force of 1600 *g* for 10 minutes at 4°C. The plasma samples were stored at −80°C until analyses were performed. The handling of human samples was performed in compliance with Peking University First Hospital's human tissue handling guidelines to avoid any risk of infection or contamination.

The following complement components were analyzed: CFB, CFH, C1q, MBL, C3, C3c, C4, C3a, C5a, and soluble C5b‐9 (sC5b‐9). For non‐pregnant women, peripheral blood samples were collected before or after their menstrual period to assess the levels of the factors mentioned above.

The plasma concentrations of CFB, CFH, C1q, C3c, and C4 were measured using immunoturbidimetric assays (BeiJia, Shanghai, China) according to the manufacturer's instructions. The plasma concentrations of C3 were measured using immunoturbidimetric assays (Beckman Coulter) according to the manufacturer's instructions. Plasma MBL levels were determined using a previously described method,^27^ with slight modifications. Mouse monoclonal MBL‐specific antibodies (HYB131‐01; Antibody Shop) at a concentration of 1 µg/mL were coated onto a microtiter plate overnight at 4°C. After blocking with 1% BSA in PBS, the plasma samples and standards were added, after which mouse monoclonal MBL‐specific antibodies (HYB131‐01b; Antibody Shop) were added. Following incubation, streptavidin‐HRP (DY998; RD) was added to the plates, which were incubated for 0.5 hour at 37°C. The reaction was developed using a tetramethylbenzidine (TMB) liquid substrate system. The plasma concentrations of C3a, C5a, and sC5‐9 were determined by enzyme‐linked immunosorbent assays (Quidel Corporation) according to the manufacturer's instructions. All these assays were performed in an accredited clinical laboratory at our hospital according to standard procedures.

The levels for each sample were calculated using CurveExpert 1.3. The linear portion of the curve was subsequently used for the measurement of plasma complement components. All assays were performed in duplicate, and samples were routinely reanalyzed when the standard error exceeded 10%.

### 2.4 Statistical analysis

SPSS 11.0 (SPSS, Chicago, IL, USA) software was employed for statistical analysis. The normality of continuous variables was assessed using the Shapiro‐Wilk test. Data are presented as the mean ± SD if normally distributed or the median if a skewed distribution was observed. To compare continuous variables among multiple groups, either ANOVA (normal distribution) or the Kruskal‐Wallis test (non‐normal distribution) was applied. The data for MBL, C3a, and sC5b‐9 levels with non‐normal distribution were analyzed after log transformation. Significant differences between the two groups were determined with the independent t test (normal distribution) or the Mann***‐***Whitney *U* test (non‐normal distribution). Covariance analysis was used to correct the effect of body mass index (BMI) on the results. Spearman rank‐order correlation was applied to calculate correlation coefficients. For all statistical analyses, *P* < .05 was considered statistically significant.

### 2.5 Study approval

This prospective study was approved and monitored by the Institutional Review Board of Peking University Health Science Center. All procedures involving human participants were performed in accordance with the ethical standards of the institutional and/or national research committee and with the 1964 Helsinki declaration and its later amendments or comparable ethical 433 standards. Written informed consent was obtained from all participants included in the study.

## 3 RESULTS

### 3.1 Patient characteristics

A total of 362 patients were eligible to participate in this study; 733 plasma samples were collected. Of the 362 patients, one plasma specimen was available for analysis for 124 (34.3%); two, three, four, and five plasma specimens were available for 132 (36.5%), 83 (22.9%), 19 (5.2%), and 4 (1.1%) patients. Because three or fewer specimens were available for most women, we analyzed the data largely in a cross‐sectional manner using all available specimens within intervals of gestational age. For 7‐week intervals, the numbers of specimens were as follows: 6‐12^+6^ weeks, 196; 13‐19^+6^ weeks, 115; 20‐27^+6^ weeks, 184; 28‐35^+6^ weeks, 120; and 36^+^ weeks, 118. A total of 65 non‐pregnant women participated in this study, with 65 plasma samples collected. The baseline clinical data at the times of enrollment and the pregnancy outcomes of all participants are shown in Table 1.

**Table 1:** Clinical characteristics of normal pregnant and non‐pregnant women at enrollment

	n	Age (y)^a^	GA at enrollment (wk)^a^	Primigravida no. (%)	BMI (kg/m^2^)	Current smoker no. (%)	GA at delivery (wk)
Pregnant women	362	31.1 ± 3.8	6.8 ± 1.2	320 (88.4%)	23.5 ± 4.6	7 (1.9%)	39.5 ± 1.1
Non‐Pregnant women	65	31.2 ± 2.2	—	—	22.6 ± 3.7	0	—
*P*		>.05	—	—	>.05	—	—

Abbreviation: GA, gestational age.

a Mean ± SD.

### 3.2 Circulating levels of CFB, CFH, C1q, and MBL in normal pregnancy

In normal pregnancy, the levels of CFB and CFH began to rise during the first trimester, continued to rise through the second trimester (weeks 20‐28), and remained stable thereafter. During 6‐12^+6^ weeks of normal pregnancy, CFB levels were 414.5 ± 85.9 mg/L (95% CI for mean: 402.4‐426.6 mg/L), and CFH levels were 381.0 ± 89.0 mg/L (95% CI for mean: 368.5‐393.6 mg/L).

The level of C1q in the peripheral circulation of pregnant women was largely unchanged compared with that in non‐pregnant women and remained stable throughout pregnancy. The level of C1q was 202.6 ± 42.4 mg/L (95% CI for mean: 196.6‐208.5 mg/L) during 6‐12^+6^ weeks of normal pregnancy.

Although the level of MBL increased significantly during the first trimester and remained stable afterward, it varied significantly from patient to patient. MBL levels were 4274.5 ± 2752 ng/mL (95% CI for mean: 3881.1‐4656.4 ng/mL) during 6‐12^+6^ weeks of normal pregnancy.

**Figure 2:**
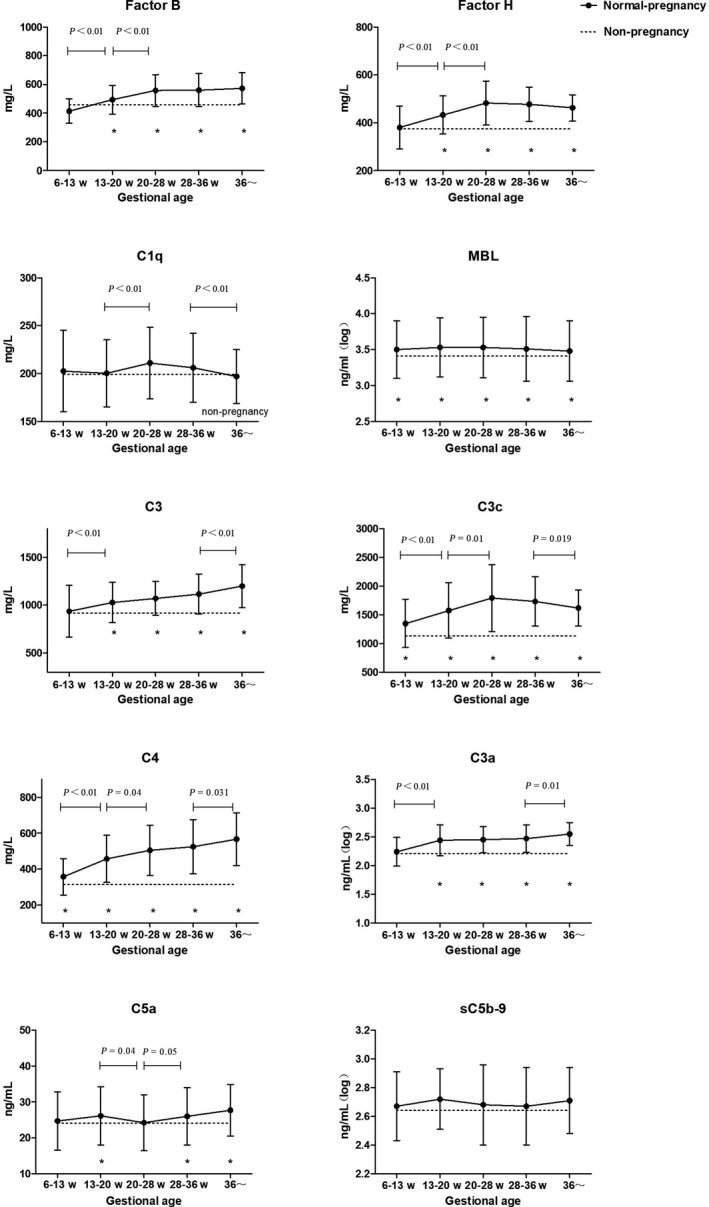
Levels of complement components in normal pregnant women

In the second and third trimester, the BMI of patients in normal pregnancy group was significantly higher than that of non‐pregnant women. Therefore, we used covariance analysis to correct the difference in BMI between the two groups, after which we found that the contents of CFB, CFH, and MBL in normal pregnancy group were significantly higher than in non‐pregnant women, all the *P* values were <.002. The levels of CFB, CFH, C1q, and MBL in women with normal pregnancy and non‐pregnant women are shown in Figure 2 and Table 2.

**Table 2:** Plasma levels of CFB, CFH, C1q, and MBL in normal pregnant and non‐pregnant women

	6‐12^+6^ wk	13‐19^+6^ wk	20‐27^+6^ wk	28‐35^+6^ wk	36 wk~	*P* a	*P* b	*P* c	*P* d
Factor B (mg/L)									
Mean ± SD	414.5 ± 85.9	493.5 ± 100.5	557.5 ± 111.5	560.8 ± 116.1	573.1 ± 108.4	<.01	<.01	.81	.40
95% CI	402.4‐426.6	474.9‐512.1	541.3‐573.7	539.8‐581.8	553.3‐592.8				
Min, max	203.9, 684.3	288.9, 941.2	295.5, 1010.9	335.6‐1080.9	406.0, 883.1				
*95% CI	246.1‐582.9	296.5‐690.5	339.0‐776.0	333.2‐788.4	360.6‐785.6				
Non‐pregnant women	458.8 ± 155.3								
*P* *	.24	<.02	<.01	<.01	<.01				
Factor H (mg/L)									
Mean ± SD	381.0 ± 89.0	432.7 ± 80.2	482.1 ± 91.2	477.2 ± 72.2	462.0 ± 54.3	<.01	<.01	.63	.07
95% CI	368.5‐393.6	417.9‐447.5	468.8‐495.3	464.2‐490.3	452.1‐471.9				
Min, max	180.8, 699.5	266.1‐666.8	306.7‐822.3	319.8, 692.1	308.3, 618.1				
*95% CI	206.6‐555.4	275.5‐589.9	303.3‐660.9	335.7‐618.7	355.6‐568.4				
Non‐pregnant women	374.1 ± 73.9								
*P* *	.45	<.01	<.01	<.01	<.01				
C1q (mg/L)									
Mean ± SD	202.6 ± 42.4	200.3 ± 35.0	211.0 ± 37.6	206.1 ± 36.1	196.9 ± 28.2	0.62	0.01	0.26	0.03
95% CI	196.6‐208.5	193.8‐206.7	205.5‐216.5	199.6‐212.7	191.7‐202.0				
Min, max	87.5, 318.1	132.3, 339.5	116.4, 358.0	135.8, 348.3	119.8, 302.6				
*95% CI	119.5‐285.7	131.7‐268.9	137.3‐284.7	135.3‐276.9	141.6‐252.2				
Non‐pregnant women	199.4 ± 35.4								
*P* *	.92	.92	.29	.64	.64				
MBL (ng/mL)									
Median ± SD	4274.5 ± 2752	4832.0 ± 2929	4251.5 ± 3182	4295.4 ± 3680	4130.5 ± 3094	.18	.96	.97	.43
95% CI	3881.1‐4656.4	4173.5‐5255.9	4270.9‐5196.6	4199.3‐5529.8	3771.4‐4899.4				
Min, max	267.5, 11 692.7	285.4, 11 826.0	194.8, 12 367.1	299.6, 17 907.8	290.7, 11 854.7				
Non‐pregnant women	2388.3 ± 2097								
*P* *	<.01	<.01	<.01	<.01	<.01				

Note

After correction for the difference in BMI, the contents of CFB, CFH, and MBL in the second and third trimester of normal pregnancy were significantly higher than in non‐pregnant women, all the *P* values were <.02.

95% CI, 95% confidence interval for mean; *95% CI, 95% confidence interval.

a 6‐12^+6^ wk vs 13‐19^+6^ wk.

b 13‐19^+6^ wk vs 20‐27^+6^ wk.

c 20‐27^+6^ wk vs 28‐35^+6^ wk.

d 28‐35^+6^ wk vs 36 wk~.

* Normal pregnancy vs non‐pregnancy.

### 3.3 Circulating levels of C3, C3c, C4, C3a, C5a, and sC5b‐9 in normal pregnancy

While measuring C3, the remaining plasma of some samples was not enough for detection. The numbers of specimens for C3 detection were as follows: 6‐12^+6^ weeks, 150; 13‐19^+6^ weeks, 87; 20‐27^+6^ weeks, 143; 28‐35^+6^ weeks, 87; and 36^+^ weeks, 80. Levels of C3 began to rise in the second trimester. For women with normal pregnancy, the peripheral level of C3 continued to rise throughout pregnancy. Levels of C3c and C4 began to rise in the first trimester. For women with normal pregnancy, the peripheral level of C3c continued to rise throughout the second trimester and then remained stable but decreased in the third trimester; the level of C4 continued to rise throughout pregnancy. During 6‐12^+6^ weeks of normal pregnancy, C3 levels were 935.5 ± 270.1 mg/L (95% CI for mean: 891.8‐979.0 mg/L), C3c levels were 1346.9 ± 419.8 mg/L (95% CI for mean: 1287.7‐1406.0 mg/L), and those of C4 were 357.4 ± 101.8 mg/L (95% CI for mean: 343.0‐371.7 mg/L). After correction for the difference in BMI, the contents of C3, C3c, and C4 in the second and third trimester of normal pregnancy were significantly higher than in non‐pregnant women, all the *P* values were <.01. The C3, C3c, and C4 levels in women with normal pregnancy are shown in Figure 2 and Table 3.

**Table 3:** Plasma levels of C3, C3c, and C4 in normal pregnant and non‐pregnant women

	6‐12^+6^ wk	13‐19^+6^ wk	20‐27^+6^ wk	28‐35^+6^ wk	36 wk~	*P* a	*P* b	*P* c	*P* d
C3 (mg/L)									
Mean ± SD	935.4 ± 270.1	1028.4 ± 210.6	1071.0 ± 178.5	1115.2 ± 206.6	1199.0 ± 224.4	<.01	.108	.09	.01
95% CI	891.8‐979.0	983.5‐1073.3	1041.1‐1101.2	1071.2‐1159.3	1149.0‐1249.3				
Min, max	476.1, 1890.2	457.2, 1490.1	653.2, 1622.1	796.0, 1681.9	756.5, 1897.2				
*95% CI	581.5‐1458.1	748.2‐1456.2	779.0‐1380.0	828.4‐1554.4	890.6‐1640.5				
Non‐pregnant women	918.6 ± 174.2								
*P* *	.648	<.01	<.01	<.01	<.01				
C3c (mg/L)									
Mean ± SD	1346.9 ± 419.8	1573.6 ± 481.5	1793.2 ± 583.6	1732.6 ± 427.6	1618.3 ± 310.9	<.01	.01	.33	.02
95% CI	1287.7‐1406.0	1484.6‐1662.5	1708.3‐1878.1	1655.3‐1810.0	1561.6‐1675.0				
Min, max	643.4, 3106.5	707.1, 4301.5	907.2, 5024.3	1089.0, 3187.2	949.1, 2724.9				
*95% CI	524.1‐2169.7	629.9‐2517.3	649.3‐2937.1	894.5‐2579.7	1000.8‐2227.7				
Non‐pregnant women	1131 ± 270								
*P* *	.01	<.01	<.01	<.01	<.01				
C4 (mg/L)									
Mean ± SD	357.4 ± 101.8	457.3 ± 131.0	504.9 ± 139.4	525.3 ± 151.5	567.0 ± 145.9	<.01	.04	.23	.03
95% CI	343.0‐371.7	433.1‐481.5	484.6‐525.2	497.9‐552.7	540.4‐593.6				
Min, max	173.4, 873.4	222.1, 924.2	193.1, 994.3	243.2, 1087.3	273.1, 1100.2				
*95% CI	157.9‐556.9	200.5‐714.1	231.7‐778.1	228.4‐822.2	281.0‐853.0				
Non‐pregnant women	314.0 ± 144								
*P* *	<.01	<.01	<.01	<.01	<.01				

Note

After correction for the difference in BMI, the contents of C3 in second and third trimester were significantly higher than that in non‐pregnant women, all the *P* values were <.01. The contents of C3c and C4 in normal pregnancy group were significantly higher than in non‐pregnant women, all the *P* values were <.01. The numbers of specimens for C3 detection were as follows: 6‐12^+6^ wk, 150; 13‐19^+6^ wk, 87; 20‐27^+6^ wk, 143; 28‐35^+6^ wk, 87; and 36^+^ wk, 80.

95% CI, 95% confidence interval for mean; *95% CI, 95% confidence interval.

a 6‐12^+6^ wk vs 13‐19^+6^ wk.

b 13‐19^+6^ wk vs 20‐27^+6^ wk.

c 20‐27^+6^ wk vs 28‐35^+6^ wk.

d 28‐35^+6^ wk vs 36 wk~.

* Normal pregnancy vs non‐pregnancy.

There were no significant differences in the levels of C3a, C5a, or sC5b‐9 in pregnant women in their first trimester compared with levels in non‐pregnant women. During 6‐12^+6^ weeks of normal pregnancy, C3a levels of were 182.5 ± 150.0 ng/mL (95% CI for mean: 186.8‐229.1 ng/mL), C5a levels were 24.7 ± 8.1 ng/mL (95% CI for mean: 23.5‐25.8 ng/mL), and sC5b‐9 levels were 468.1 ± 404.4 ng/mL (95% CI for mean: 497.6‐611.6 ng/mL). The level of C3a was elevated throughout pregnancy, but that of C5a and sC5b‐9 remained largely unchanged. After correction for the difference in BMI, the level of C3a in the second and third trimesters and that of C5a in the third trimester were higher than that in healthy non‐pregnant women. The levels of C3a, C5a, and sC5b‐9 in women with normal pregnancy are shown in Figure 2 and Table 4.

**Table 4:** Plasma levels of C3a, C5a, and sC5b‐9 in normal pregnant and non‐pregnant women

	6‐12^+6^ wk	13‐19^+6^ wk	20‐27^+6^ wk	28‐35^+6^ wk	36 wk~	*P* a	*P* b	*P* c	*P* d
C3a (ng/mL)									
Median ± SD	182.5 ± 150.0	307.5 ± 175.2	280.7 ± 170.7	320.0 ± 172.1	365.5 ± 188.8	<.01	.62	.27	.01
95% CI	186.8‐229.1	292.6‐357.3	295.8‐345.5	307.1‐369.3	361.0‐429.8				
Min, max	42.6, 1429.6	26.1, 1103.5	32.9, 984.9	48.7, 923.8	108.1, 1158.6				
Non‐pregnant women	164.4 ± 94								
*P* *	.08	<.01	<.01	<.01	<.01				
C5a (ng/mL)									
Mean ± SD	24.7 ± 8.1	26.1 ± 8.1	24.2 ± 7.8	26.0 ± 8.0	27.7 ± 7.2	.14	.04	.05	.09
95% CI	23.5‐25.8	24.6‐27.6	23.0‐25.3	24.5‐27.4	26.3‐29.0				
Min, max	6.3, 39.9	9.9, 37.1	6.34, 37.0	7.2, 40.7	8.1, 42.4				
*95% CI	8.8‐40.6	10.2‐42.0	8.9‐39.5	10.3‐41.7	13.6‐41.8				
Non‐pregnant women	24.1 ± 8.6								
*P* *	.88	.045	.94	.047	.04				
sC5b‐9 (ng/mL)									
Median ± SD	468.1 ± 404.4	511.5 ± 380.2	448.8 ± 514.7	424.5 ± 668.5	459.6 ± 517.5	.16	.06	.63	.08
95% CI	497.6‐611.6	526.7‐667.2	526.2‐675.9	491.2‐732.9	513.3‐702.8				
Min, max	59.1, 3769.2	189.0, 2733.1	102.0, 2725.3	147.1, 4563.2	160.2, 3291.7				
Non‐pregnant women	562.0 ± 386.2								
*P* *	.92	.66	.63	.60	.36				

Note

After correction for the difference in BMI, the level of C3a in the second and third trimesters and that of C5a in the third trimester were higher than that in healthy non‐pregnant women.

95% CI, 95% confidence interval for mean; *95% CI, 95% confidence interval.

a 6‐12^+6^ wk vs 13‐19^+6^ wk.

b 13‐19^+6^ wk vs 20‐27^+6^ wk.

c 20‐27^+6^ wk vs 28‐35^+6^ wk.

d 28‐35^+6^ wk vs 36 wk~.

* Normal pregnancy vs non‐pregnancy

### 3.4 Circulating levels of complement components in non‐pregnant women

All the non‐pregnant women in our study were in reproductive age, as shown in Table 1, the average age of non‐pregnant women was 31.2 years. Since the levels of complement components may be different between luteal and follicular phases, we compared the levels of complement components between luteal and follicular phases.

Levels of CFB, C3c, and C4 are higher in the luteal phase than that in the follicular phase (CFB: 437.9 ± 71.3 vs 473.7 ± 190.6 mg/L, *P* < .01) (C3c: 1121.0 ± 202.5 vs 1138.6 ± 300.1 mg/L, *P* = .045) (C4: 286.2 ± 86.9 vs 333.2 ± 169.3 mg/L, *P* < .01). Levels of CFB, C3c, and C4 in the second and third trimesters of normal pregnancy were higher than those in healthy non‐pregnant women, even only compared with women in the luteal phase. The levels of complement components in follicular phase and luteal phase are shown in Table S1.

### 3.5 Correlations of C3c, C3a, and C5a in the second and third trimesters of normal pregnancy and in non‐pregnant women

**Figure 3:**
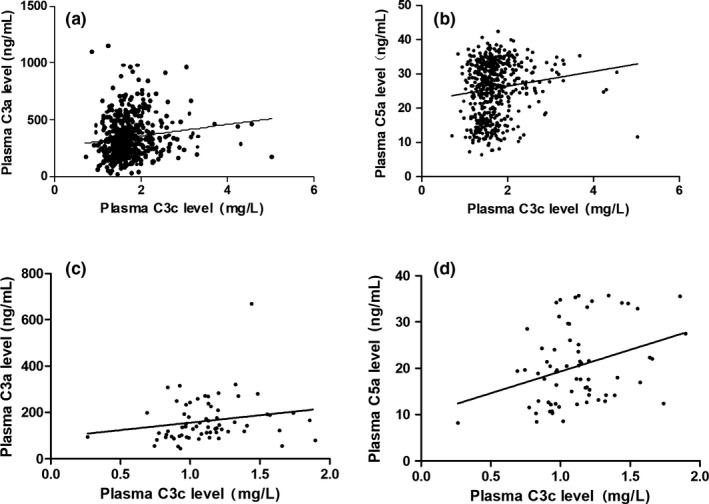
Scatterplots of C3a versus C3c and C5a versus C3c in second and third trimester in normalpregnancy (a, b) and in non‐pregnant women (c, d)

We also investigated whether plasma C3c levels are related to concentrations of C3a and C5a in the second and third trimesters in normal pregnancy and non‐pregnant women. We found that C3a showed significant positive correlation to C3c in second and third trimester in normal pregnancy but not in non‐pregnant women (for normal pregnancy: *r* = .137, *P* = .001; for non‐pregnant women: *r* = .038, *P* = .116). C5a showed significant positive correlation to C3c both in second and third trimester in normal pregnancy and in non‐pregnant women (for normal pregnancy: *r* = .133, *P* = .02; for non‐pregnant women: *r* = .103, *P* = .008). The relationships between C3c and C3a and C5a are presented in Figure 3.

## 4 DISCUSSION

Components of the complement system are important for normal placentation, and delicate regulation of complement activation is critical for successful pregnancy. Literature from approximately 50 years ago demonstrates increased circulating concentrations of C3 at term in normal pregnancy.^28^ Additionally, Baines et al^29^ investigated the circulating concentration of C3 over the continuum of pregnancy, finding an initial decrease in the first trimester and an increase in the second and third trimesters, though C3 returned to early pregnancy values after delivery. A more recent study by Derzsy et al^20^ measured circulating levels of complement components and activation products C4d, C3a, sC5b‐9 along with CFH and found that concentrations of C4d, C3a, sC5b‐9, C3, C9, and CFH were greater in normal pregnancy at 36‐37 weeks of gestation than those in non‐pregnant women. In the current study, we analyzed the plasma levels of major complement components in women with normal pregnancy during different gestational stages, and the results revealed that the complement system is activated in the second and third trimesters of normal pregnancy, as expressed by increased concentrations of C3a and C3c in the systemic circulation. There are many plausible explanations for complement activation in healthy pregnancy.^30^, ^31^, ^32^ During placental formation in the first and second trimesters, many apoptotic cells and free DNA are produced, and this cellular debris and DNA fragments can lead to moderate activation of the complement system, which facilitates the removal of cell fragments and placental formation. However, our study also found that the levels of sC5b‐9 remained largely unchanged during pregnancy compared with those in non‐pregnant women. These data suggest that excessive complement activation does not progress beyond C3 and C5 levels in normal pregnancy.

Increased circulating concentrations of Bb in the first trimester of pregnancy have been documented as an early marker of preeclampsia, suggesting that alternative pathway activation is important in the pathogenesis of preeclampsia.^33^ Nonetheless, pregnancy itself may activate the complement system via the alternative pathway. Regardless, there are a few studies on this topic, and studies on the inhibitory factor of this pathway, CFH, are particularly rare. In our study, CFB levels began to rise during the first trimester, suggesting that the alternative pathway may be involved in placental formation. Because CFH levels increased along with those of CFB and C3a, we hypothesize that the relative abundance of CFH in the second and third trimesters of normal pregnancy might prevent complement activation via the alternative pathway at the C3 level.

In our study, C3 levels were similar to that in non‐pregnant women in early pregnancy, but began to rise thereafter. C4 levels began to rise in early pregnancy and continued to rise throughout the second and third trimesters. In fact, local C4 levels at the maternal‐fetal interface also rise in normal pregnancy. A previous in vitro study^9^ showed that during the first trimester, extracellular trophoblasts can secrete C4, which may assist in trophoblast invasion of the decidua and endometrial blood vessels. The increase in C3 and C4 is mainly due to the enhanced maternal synthetic function that occurs in normal pregnancy; however, several factors can regulate circulating levels of C3 and C4 in normal pregnancy. In early pregnancy, along with the activation of complement system, the concomitant C3 consumption might result in unchanged or even decreased level of C3 in the first trimester of normal pregnancy.^20^, ^29^

Many previous studies have reported that C1q can be synthesized by macrophages and basal decidual cells at the maternal‐fetal interface or by penetrating trophoblasts.^8^ These latter cells can synthesize and secrete C1q during penetration into the basal decidua in the first trimester, and secreted C1q facilitates trophoblastic penetration into the uterine basal layer to assist in placental formation.^10^, ^34^ Moreover, C1q is an angiogenic factor that promotes neovascularization, thus ensuring a normal pregnancy.^34^ However, Bulla et al^8^ found that although C1q is important for connecting endovascular trophoblasts and decidual endothelial cells, no C4 colocalizes with C1q at the maternal‐fetal interface, suggesting that C1q does not initiate complement activation at this site. In our study, no significant increase in C1q level was observed during pregnancy compared with that in non‐pregnancy, with the level remaining stable during pregnancy. Thus, we hypothesize that changes in C1q during early pregnancy may be more localized at the maternal‐fetal interface and that the level of C1q in peripheral blood may not fully reflect the local C1q changes occurring at the maternal‐fetal interface.

Several studies suggest that maternal MBL deficiency is a risk factor for adverse pregnancy outcomes and that a high level of MBL is beneficial for pregnancy.^35^, ^36^, ^37^ In the present study, increased MBL concentrations were observed in the first trimester, and these levels did not significantly increase further during pregnancy. The observed increase in MBL during pregnancy in our study of 362 women without adverse pregnancy outcomes is consistent with an observation by van de Geijn FE,^38^ who described an increase in MBL during pregnancy in 32 women with no obstetric history. Previous studies have also shown that the level of MBL is closely related to the genotype,^39^ which may explain why we observed substantial MBL variability in the individuals enrolled in this study.

In this study, we found that C3a showed significant positive correlation to C3c in second and third trimester in normal pregnancy but not in non‐pregnant women. C5a showed significant positive correlation with C3c both in second and third trimester in normal pregnancy and in non‐pregnant women. Although the relationships are statistically significant, the relation coefficients are small and may not be clinically significant.

This study shows that activation of the complement system begins in the first trimester of a normal pregnancy and continues throughout the pregnancy. Overall, moderate complement activation may play an important role in placental formation and normal pregnancy maintenance. In normal pregnancy, complement activation does not progress beyond C3 and C5 levels, and the relative abundance of regulatory proteins, for example, CFH, may prevent excessive complement activation. The results of our study offer unique opportunities to examine regulation of the complement system in the future and its role in the pathophysiology of pregnancy.

The major limitation of this study was that although it was designed as a longitudinal study, three or more plasma samples were available for <30% of the patients, resulting in a largely cross‐sectional analysis of the data.

## 5 CONCLUSION

In normal pregnancy, complement activation begins in the first trimester and continues throughout pregnancy. Additionally, complement activation does not progress beyond C3 and C5 levels, and the relative abundance of CFH may prevent excessive complement activation in normal pregnancy.

## CONFLICT OF INTEREST

None of the authors received any support for this study. On behalf of all authors, the corresponding author states that there is no conflict of interest.
