# Supplementary material for: Normal range of complement components during pregnancy: A prospective study
Source: Am J Reprod Immunol. 2019 Nov 12;83(2):e13202. doi: 10.1111/aji.13202 (PMC7027513; doi:10.1111/aji.13202)
Supplement: Supplementary file 1 [file AJI-83-e13202-s001.docx]

Supplementary table 1. Levels of complement components in follicular phase and luteal phase.

|  | Follicular phase (n=26) | Luteal phase (n=39) | P |
| --- | --- | --- | --- |
| CFB（mg/L） | 437.9±71.3 | 473.7±190.6 | ＜0.01 |
| CFH（mg/L） | 373.0±64.3 | 374.8±79.7 | 0.277 |
| C1q（mg/L） | 204.6±27.8 | 195.8±39.2 | 0.078 |
| MBL（ng/ml） | 1738.9±2022. | 2138.7±2131 | 0.366 |
| C3（mg/L） | 907.1±164.1 | 936.5±191.3 | 0.518 |
| C3c（mg/L） | 1121.0±202.5 | 1138.6±300.1 | 0.045 |
| C4（mg/L） | 286.2±86.9 | 333.2±169.3 | ＜0.01 |
| C3a（ng/ml） | 135.7±61.5 | 142.1±110.6 | 0.93 |
| C5a（ng/ml） | 21.2±8.0 | 20.2±8.3 | 0.884 |
| sC5b-9（ng/ml） | 472.4±426.6 | 475.2±357.2 | 0.815 |

CFB, CFH, C1q, C3, C3c, C4, C5a: Mean±SD; C3a, sC5b-9: Median±SD

Levels of CFB, C3c, and C4 are higher in the luteal phase than that in the follicular phase.
